# Structural Elucidation and Toxicity Assessment of Degraded Products of Aflatoxin B1 and B2 by Aqueous Extracts of *Trachyspermum ammi*

**DOI:** 10.3389/fmicb.2016.00346

**Published:** 2016-03-30

**Authors:** Wajiha Iram, Tehmina Anjum, Mazhar Iqbal, Abdul Ghaffar, Mateen Abbas

**Affiliations:** ^1^Institute of Agricultural Sciences, University of the PunjabLahore, Pakistan; ^2^Health Biotechnology Division, National Institute for Biotechnology and Genetic EngineeringFaisalabad, Pakistan; ^3^Department of Chemistry, University of Engineering and TechnologyLahore, Pakistan; ^4^Department of Toxicology, Quality Operating Laboratory, University of Veterinary and Animal SciencesLahore, Pakistan

**Keywords:** aflatoxin, degradation, plant extract, LCMS/MS, toxicity

## Abstract

In this study aqueous extract of seeds and leaves of *Trachyspermum ammi* were evaluated for their ability to detoxify aflatoxin B1 and B2 (AFB1; 100 μg L^−1^ and AFB2; 50 μg L^−1^) by *in vitro* and *in vivo* assays. Results indicated that *T. ammi* seeds extract was found to be significant (*P* < 0.05) in degrading AFB1 and AFB2 i.e., 92.8 and 91.9% respectively. However, *T. ammi* leaves extract proved to be less efficient in degrading these aflatoxins, under optimized conditions i.e., pH 8, temperature 30°C and incubation period of 72 h. The structural elucidation of degraded toxin products by LCMS/MS analysis showed that eight degraded products of AFB1 and AFB2 were formed. MS/MS spectra showed that most of the products were formed by the removal of double bond in the terminal furan ring and modification of lactone group indicating less toxicity as compared to parent compounds. Brine shrimps bioassay further confirmed the low toxicity of degraded products, showing that *T. ammi* seeds extract can be used as an effective tool for the detoxification of aflatoxins.

## Introduction

Mycotoxins are chemically and biologically active secondary metabolites produced by fungi in cereals, nuts, fruits and vegetables (Sinha and Sinha, 1991; Aly, 2002). About 25% of the world cereals are contaminated with known mycotoxins produced by variety of toxigenic fungi. The food and agriculture organization (FAO) estimates that about 1000 million metric tons of foodstuffs could be contaminated with mycotoxins each year (Bhat et al., 2010). Currently, more than 400 mycotoxins are identified, among them, aflatoxins are the most serious carcinogenic, hepatotoxic, teratogenic, and mutagenic secondary metabolites which adversely affect humans and animal health. They are classified as group-1 carcinogens by International Agency for Research on cancer (IARC, 2002). Aflatoxin contamination can occur at any stage of food production from pre-harvest to storage (Wilson and Payne, 1994). Factors that affect aflatoxin contamination include the climate, genotype of the crop planted, soil type, temperature fluxes, intercropping with infected grains, early and delayed harvesting, improper drying and meager storage conditions (Folayan, 2013).

Aflatoxins are biologically active polyketide derived secondary metabolites which consist of a group of closely related highly oxygenated bisfurano-coumarin heterocyclic compounds, mostly produced by *Aspergillus flavus* and *Aspergillus parasiticus* (Ellis et al., 1991; Bhatnagar et al., 1992). The G series of aflatoxins differs chemically from B series by the presence of a β lactone ring, instead of cyclopentenone ring. In AFB1 and AFG1 a double bond undergoes reduction forming vinyl ether at the terminal furan ring but not in AFB2 and AFG2 (Samarajeewa et al., 1990). AFB1 and AFG1 are carcinogenic and considerably more toxic than AFB2 and AFG2 probably due to these small difference in structure (Jaimez et al., 2000).

Various physical, chemical and biological methods have been described for detoxification of mycotoxins. Routinely, physical and chemical methods like roasting, flaking, canning, alkalization, oxidation, reduction and acidification are being frequently used. But these methods have not yet proven to be as effective and desirable because they are considered to be potentially unsafe as some form toxic residues or even alter the nutritional contents and flavor of treated commodity. In addition these methods require sophisticated equipment rendering for their high cost and environmental pollution (Joseph et al., 2005; Shukla et al., 2012). So, health hazards from exposure to such methods and economic consideration make biological control and natural plants extracts ideal alternatives to protect food and feed from fungal and mycotoxin contamination as they are biologically safe, eco-friendly, cheap, easily available, lack residual effects and are easily degradable (Reddy et al., 2007).

For the last decade, the use of herbal food additives has been encouraged (Mirzaei-Aghsaghali, 2012). The intensive efforts have been made by various researches for the clarification of biochemical structures and physiological functions of various food and feed additives like prebiotics, probiotics, organic acids and plant extracts. Numerous aromatic plants have been found to inhibit the microbial growth and thereby traditionally used to extend the shelf life of food (Ahmed et al., 2014). Similarly, several medicinal herbs and spices have been reported to counteract deleterious effects of mycotoxins either by chemical modification or by inclusion into the plant matrix (Wallnofer et al., 1996). Powder and extract of many medicinal herbs and higher plants have been shown inhibitory effect on growth of toxigenic fungi and production of toxins (Solis et al., 1993; Momoh et al., 2012). Based on the literature following plants were found to be effective in detoxifying aflatoxins and growth inhibition of toxigenic fungi: *Withania somnifera* (Linn.), *Camellia sinensis* (Linn.), *Citrus medica* (Linn.), *Syzigium aromaticum* (L.) Merr. Et Perry, *Curcuma longa* (L.), *Allium sativum* L. and *Ocimum sanctum* (Linn.), *Trachyspermum ammi* (L.), *Eucalyptus globolus* (Linn.), *Olea europaea* (Linn.), *Thymus vulgaris, Hibiscus sabdariffa* (Linn.), *Boswellia sacra, Adhatoda vasica* Nees and *Barleria lupulina* Lindl (Krishnamurthy and Shashikala, 2006; Reddy et al., 2009; Velazhahan et al., 2010; Al-Rahmah et al., 2011; El-Nagerabi et al., 2012, 2013; Kannan and Velazhahan, 2014; Vijayanandraj et al., 2014).

In the present investigation, *Trachyspermum ammi* (Family: Apiaceae, Common name: Ajwain) was used to evaluate its aflatoxin detoxifying potential. *T. ammi* has been known to possess known antimicrobial, antispasmodic, antiflatulent, antioxidant and antirheumatic effect due to the presence of several active compounds (Bairwa et al., 2012). Phytochemical studies on *T. ammi* revealed the presence of alkaloids, phenolics, steroids, fixed oils, glycosides, tannins, saponin and flavonoids, cumene, thymene, amino acids and thymol (Asifa et al., 2014). Literature showed that phenols, thymol and carvacol, are responsible for its antimicrobial, anti mycotoxigenic, antiseptic, antitussive and expectorant properties (Pathak et al., 2010). Ajwain is generally regarded as safe when taken in the recommended doses, however, in rare cases, it can cause nausea and headache (Grossberg and Fox, 2008). Although previous studies have been conducted with *T. ammi* seeds extract to detoxify aflatoxins but their resulting degradation products are not described in detail. However, in the present manuscript structural identification and fragmentation patterns of proposed degradation products were included along with their toxicity assessment. Various parameters were optimized for *in vitro* and *in vivo* detoxification of aflatoxins.

## Materials and methods

### Extraction and purification of aflatoxins

Aflatoxin B1 and B2 were extracted from a toxigenic isolate of *Aspergillus flavus* (isolated from stored maize samples) grown on coconut cream media by solvent extraction method as described by Yazdani et al. (2010) with some modifications. For extraction, colony margins were scraped together with surrounding zones into 250 mL Erlenmeyer flask containing 10 mL chloroform: acetone (85:15 v/v) and shacked for 30 min at 200 rpm. The crude extracts were filtered through gauze, and then through Whatman No.1 filter paper. Then, the filtrate was passed through the immunoaffinity column (Aflatest column, VICAM, Waters, USA) in solid phase extraction assembly for the separation of aflatoxins, according to the method described by Stroka et al. (2000) with some modifications. The column was rinsed with HPLC grade water and 10 ml of the sample was passed through it. Based on the highly specific antibody antigen reaction, the aflatoxins present in the sample form a conjugate with the antibody and the remaining impurities are separated out. Finally aflatoxins bound in the column are cleaved from their respective antibodies using methanol and compared with standard AFB1 and AFB2 purchased from (Sigma-Aldrich, St. Louis, MO, USA) through High Performance Liquid Chromatography. Stock solutions of AFB1 (1000 μg L^−1^) and AFB2 (500 μg L^−1^) were prepared in methanol and stored at 4°C. The working solutions of AFB1 (100 μg L^−^) and AFB2 (50 μg L^−1^) were prepared by diluting the stock solution.

### Preparation of plant extract

Plant extracts were prepared according to the method described by Velazhahan et al. (2010) with some modifications. Samples were surface-sterilized using 1% sodium hypochlorite for 10 min and washed several times with sterile distilled water. After that aqueous extract of *Trachyspermum ammi* leaves and seeds was prepared by homogenizing 10 g of leaves/seeds with 10 mL of sterile distilled water. Homogenate was filtered through muslin cloth and centrifuged at 14,000 rpm for 20 min. Supernatant was sterilized using syringe filter assembly and used for further detoxification studies.

### *In vitro* toxin inactivation assay

For detoxification studies, 50 μL of working solution containing (100 μg L^−1^) AFB1 and (50 μg L^−1^) AFB2 was mixed with 250 μL of *T. ammi* plant extracts and incubated for various intervals of time. After incubation, 250 μL of Chloroform was added to above mixture and mixed well by vortexing. After that, the mixture was centrifuged at 13,000 rpm for 10 min in a centrifuge (Eppendorf, 5424C) to separate the chloroform fraction. After centrifugation, the chloroform fraction was transferred to another glass tube, evaporated to dryness under gentle stream of nitrogen and re-dissolved in methanol. Control consisted of 50 μL of toxin in 250 μL of water and was incubated under same conditions. All experiments were conducted in triplicate.

### *In vitro* optimization of parameters for toxin detoxification

#### pH

The optimal pH was determined by modifying the original pH of the *T. ammi* aqueous extracts in the range of 2.0–10.0 (adjusted using either 1 N HCl or 1 N NaOH) and then assayed for toxin detoxification activity. Distilled water with same pH range as well as untreated extract was used as control.

#### Temperature and incubation time

For assessing optimum temperature and incubation period, *T. ammi* extracts were incubated with toxins at 25°C, 30°C, 35°C, 40°C, 45°C, 50°C, 55°C, and 60°C for 3, 6, 12, 24, 48, and 72 h respectively. After incubation, the toxin content in the reaction mixture was determined as described above.

### Effect of boiling on toxin detoxification properties of plants extracts

In order to study the effect of boiling on toxin detoxification properties of plants extracts, 1mL of aqueous plant extract was added in 1.5 mL eppendorf tube and placed in a boiling water bath for 5–10 min, cooled to room temperature and then tested for toxin detoxification activity.

### Detoxification of maize samples using plant extracts (*In vivo* studies)

In *In vivo* studies, ten grams of maize seeds were kept in each 250 ml Erlenmeyer flask and spiked with 3ml of aflatoxins (with concentration B1 100 μg L^−1^ and B2 50 μg L^−1^) according to the method described by Das and Mishra (2000) with some modification. These samples were then incubated with 10 ml of *T. ammi* seeds and leaves aqueous extract at 30°C for 72 h.

After incubation, aflatoxins extraction was performed according to the method described by Stroka et al. (2000) with some modifications. Maize samples were extracted with water–acetonitrile (15: 85 v/v) and incubated on shaking water bath for 2 h. After incubation, the extracts were filtered through filter paper (Whatman, Inc., Clifton, NJ, USA). Immunoaffinity columns were conditioned with double distilled water. Then, the filtrate was passed through the column in a solid phase extraction assembly. Toxins were slowly eluted from the column with 1mL methanol in a glass vial. The residual toxin was qualitatively and quantitatively analyzed by TLC and HPLC respectively. Controls consist of untreated maize sample, sample with toxin without *T. ammi* extract, sample with *T. ammi* extract without toxin. Experiments were done in triplicate.

### Detection and quantification of treated toxin

The detection and qualification of residual toxin was determined by thin layer chromatography (TLC) according to the method described by Ramesh et al. (2013) with some modifications. Twenty microliters of chloroform methanol fraction of treated and control samples were spotted on 0.25 mm silica gel 60F_254_ (20 × 20 cm, Merck) TLC plate and developed in chloroform: acetone (92:8 v/v). The developed plates were viewed under UV light at 365 nm.

Quantitative analysis of treated and untreated toxin was done by using High Performance Liquid Chromatography (HPLC) after derivatization. Derivatization was carried out as described by Hernandez-Hierro et al. (2008) with some modifications. For this purpose, elute was evaporated to dryness with gentle stream of nitrogen, redissolved in 200 μL of n-Haxane, vortexed for 30 s. Next, 50 μL of trifluroacetic acid (TFA) was added to it. Finally, 950 μL of acetonitrile-water (1:9) was added to above solution and filtered by using syringe filter assembly. The filtrate was analyzed by HPLC.

A HPLC system (Agilent 1100 series, Agilent Technologies, Santa Clara, CA, USA) with a reversed- phase C18 column (Merck, Darmstadt, Germany) and a fluorescence detector was used for quantification. Mobile phase consisting of water: methanol: acetonitrile in the volume ratio 60:20:20 at a flow rate of 1 mL/min was applied and aflatoxin was detected at excitation and emission wavelengths of 360 and 440 nm respectively. For HPLC method validation, calibration curves were drawn using a series of calibration solutions in methanol. Each standard solution was chromatographed in duplicate. Further, identification of degraded toxin metabolites was carried out by mass spectral studies.

### LCMS analysis of degraded toxin

Toxin products were analyzed by using surveyor LC system equipped with mass spectrophotometer and PDA plus detectors (Thermo Fisher Scientific). The system was validated with known standards individually and in mixture form. All analysis were performed in triplicate using luna phenomenex C_18_ column (150 × 4.6 mm, 3 μm), in isocratic mode. Following are the LC-MS conditions for Aflatoxins. Injection volume was 10 μL. The mobile phase consisted of Methanol: Acetonitrile: Water (22.5: 22.5: 55.0 v/v). Column temperature was maintained at 30°C. The total operation time was 25 min with the flow rate of 0.5 mL min^−1^. MS conditions were as follows: capillary temperature was 335°C, sheath gas flow and Auxiliary gas flow was 20 L min^−1^ and 4 L min^−1^ respectively. Source voltage, capillary voltage and tube lens voltage was 5 KV, 49 V, and 120 V respectively. Toxins incubated with water instead of plant extracts, under optimized conditions of pH (8.0) and temperature (30°C) were run as control in LCMS analysis.

### ESI—MS/MS conditions for aflatoxins through direct insertion pump

Samples were further analyzed by mass spectrometer with electrospray ionization (ESI) to predict the molecular formulae as well as elemental composition of degraded products of AFB1 and AFB2. Mass spectrometery/Mass spectrometery was performed on a Thermo Scientific LTQ XL System fitted with electrospray ionization (ESI) source operating in positive ionization mode with optimum conditions set as follows: capillary voltage to 49.0 V, source voltage to 5.0 KV, Tube lens voltage to 110 V, and capillary temperature to 275°C. Sheath and auxiliary gas flow were adjusted to get stable spray i.e., 3 L min^−1^ and 0.4 L min^−1^ respectively. Data were collected in positive mode within the range of 100–500 m/z. The final identification of an unknown compound was based on the accurate mass measurement of parent and fragments ions, as well as other useful MS/MS spectrum information (Wang et al., 2011). Untreated toxins (AFB1 and AFB2) and water treated toxins were run as control in MS/MS experiments.

### Testing biological toxicity of degraded products

The biological toxicity of degraded toxin products was tested using brine shrimps (*Artemia salina*) bioassay. The procedure for the bioassay generally followed the method developed by Solis et al. (1993) with some modifications. Brine shrimps dry eggs were procured from local market. 100–200 mg of shrimps eggs were hatched in artificial sea water (34 g sea salt/L of deionized water) by incubation under 60 W lamp, providing direct light and warmth (26°C). After an incubation period, the hatched nauplii were separated from shells and transferred to fresh sea water.

300 μl of treated and untreated AFB1 (100 μg L^−1^) and AFB2 (50 μg L^−1^) solution was added to 96 well plate separately and dried overnight. After complete evaporation of solvent, toxins were re-dissolved in 200 μL of sea water. 200 μL of sea water containing 40–45 organisms were pipetted into each well, resulting in a final volume of 400 μL and incubated for 24–96 h at 26°C. Mortality was determined by counting the immobile (dead) larvae under stereoscope microscope. Toxicity of each solution was evaluated in triplicate.

### Statistical analysis

Results obtained in various experiments were subjected to statistical analysis by using DSSTAT software. Data were analyzed by analysis of Variance (ANOVA) and differences among the means were determined for significance at *P* ≤ 0.05 using Tukey's multiple range test.

## Results

### Effect of temperature and incubation period on toxin detoxification by *T. ammi* extracts (*In vitro*)

Aqueous extracts of *Trachyspermum ammi* leaves and seeds were evaluated for their ability to detoxify aflatoxin B1 and B2 at different temperatures and incubation time. The extent of detoxification was compared with that of control under same conditions. Time course study of toxin degradation showed that detoxification of AFB1 and AFB2 started within 3 h of incubation and percentage of degradation increased with increase in incubation time (Table 1). Results indicated that at lowest tested temperature of 25°C, aqueous extract of *T. ammi* seeds showed higher detoxification of AFB1 and AFB2 after 3 h of incubation i.e., 52.4 and 69.3% respectively as compared to *T. ammi* leaves extract. This percentage of detoxification increased with increase in incubation time to 72 h that detoxified AFB1 to 79.8% and AFB2 up to 78.9%.

**Table 1:** **Effect of Temperature and Incubation Period on AFB1 and AFB2 Detoxification by Plant Extracts**.

**Control**	**Temp (°C)**	**AFB1 percentage reduction**						**AFB2 percentage reduction**					
		**3 h**	**6 h**	**12 h**	**24 h**	**48 h**	**72 h**	**3 h**	**6 h**	**12 h**	**24 h**	**48 h**	**72 h**
**Aflatoxin**	25	0.25^d I^ (1.64)	0.71^cd H^ (1.86)	1.45^bc J^ (0.84)	1.71^b H^ (1.65)	1.94^b H^ (0.42)	2.86^a I^ (1.54)	0.12^c M^ (0.14)	0.53^bc M^ (1.63)	0.72^abc N^ (1.38)	0.84^abc L^ (1.89)	1.02^ab M^ (1.72)	1.44^a N^ (1.34)
	30	0.80^d I^ (1.98)	0.80^d H^ (1.98)	1.88^cd J^ (1.44)	2.30^bc H^ (1.32)	3.07^ab GH^ (1.32)	3.81^a I^ (1.04)	0.17^c M^ (1.23)	0.68^bc M^ (1.73)	0.79^bc N^ (1.62)	0.99^ab L^ (0.73)	1.16^ab M^ (1.11)	1.66^a N^ (1.20)
	35	1.21^c I^ (0.96)	2.54^bc H^ (1.65)	2.54^bc J^ (1.53)	3.01^abc GH^ (1.08)	3.80^ab GH^ (1.81)	4.49^a HI^ (1.76)	0.23^c M^ (1.16)	0.82^bc M^ (0.81)	0.87^bc N^ (1.37)	1.14^b L^ (1.08)	1.31^ab M^ (1.21)	1.88^a N^ (1.15)
	40	2.21^b I^ (1.85)	3.53^ab H^ (1.24)	3.87^ab IJ^ (1.39)	4.33^a GH^ (1.73)	4.46^a FGH^ (1.93)	5.15^a GHI^ (1.34)	0.28^c M^ (1.87)	0.94^bc M^ (1.71)	0.97^bc N^ (1.78)	1.29^b L^ (1.87)	1.46^ab M^ (0.77)	2.11^a MN^ (1.52)
	45	3.20^b I^ (1.31)	4.53^ab H^ (1.98)	5.13^a IJ^ (1.88)	5.19^a GH^ (0.49)	5.66^a FGH^ (1.47)	5.82^a GHI^ (1.72)	0.33^c M^ (1.28)	1.02^bc M^ (1.38)	1.12^b N^ (1.24)	1.44^b L^ (0.96)	1.61^b M^ (1.81)	2.33^a MN^ (0.85)
	50	4.19^b I^ (1.57)	5.52^ab H^ (0.74)	5.79^ab IJ^ (1.97)	6.48^a GH^ (1.24)	6.51^a FGH^ (0.48)	6.98^a GHI^ (0.27)	0.38^c M^ (1.68)	1.09^bc M^ (0.28)	1.27^b N^ (1.56)	1.59^b L^ (1.26)	1.76^b M^ (0.56)	2.55^a MN^ (1.28)
	55	5.18^c I^ (1.76)	6.45^bc H^ (1.92)	6.51^bc IJ^ (1.31)	7.14^ab GH^ (1.21)	7.84^ab FGH^ (1.25)	8.30^a GHI^ (1.64)	0.43^d M^ (0.92)	1.16^cd M^ (1.42)	1.42^bc N^ (1.07)	1.73^bc L^ (1.37)	1.91^b M^ (1.29)	2.78^a MN^ (1.17)
	60	6.18^c I^ (1.65)	7.11^c H^ (1.34)	7.50^c IJ^ (1.14)	7.80^bc GH^ (1.30)	9.16^ab FGH^ (1.64)	9.63^a GHI^ (1.03)	0.49^d M^ (1.72)	1.24^cd M^ (1.87)	1.57^bc N^ (1.81)	1.88^bc L^ (1.28)	2.06^b M^ (1.54)	3.00^a MN^ (1.89)
**Aflatoxin + Water**	25	0.28^b I^ (0.62)	1.48^b H^ (1.46)	2.72^ab J^ (1.12)	3.35^a GH^ (1.78)	3.36^a GH^ (1.54)	3.38^a I^ (1.17)	0.29^c M^ (1.78)	0.47^c M^ (1.21)	1.32^bc N^ (0.47)	1.47^abc L^ (1.62)	2.25^ab M^ (1.74)	2.46^a MN^ (1.12)
	30	1.10^c I^ (1.35)	2.64^bc H^ (1.82)	3.23^ab J^ (0.89)	3.56^ab GH^ (1.85)	3.97^ab FGH^ (1.34)	4.19^a HI^ (0.81)	0.36^c M^ (1.19)	1.14^bc M^ (1.91)	1.56^bc N^ (1.18)	2.04^ab L^ (1.27)	2.41^ab M^ (0.37)	3.41^a MN^ (1.23)
	35	2.44^c I^ (1.54)	3.44^bc H^ (0.99)	4.80^ab IJ^ (1.56)	5.09^a GH^ (0.65)	5.49^a FGH^ (0.37)	5.85^a GHI^ (0.53)	1.20^c M^ (1.33)	1.23^c M^ (1.02)	2.26^b MN^ (1.72)	2.73^ab KL^ (0.18)	2.87^ab M^ (1.29)	3.34^a MN^ (0.62)
	40	3.76^c I^ (1.98)	4.76^bc H^ (1.02)	6.13^ab IJ^ (1.44)	6.48^a GH^ (1.21)	6.74^a FGH^ (1.72)	6.84^a GHI^ (1.71)	1.98^c LM^ (1.27)	2.69^bc M^ (1.75)	3.37^ab MN^ (1.62)	3.40^ab KL^ (1.29)	3.71^a M^ (1.13)	3.84^a MN^ (1.91)
	45	5.08^c I^ (0.87)	6.08^bc H^ (1.16)	7.45^ab IJ^ (0.81)	7.47^ab GH^ (0.45)	7.83^a FGH^ (1.11)	8.40^a GHI^ (0.67)	2.72^c KLM^ (1.54)	3.92^b M^ (1.87)	4.09^ab MN^ (0.31)	4.18^ab KL^ (1.89)	4.49^ab M^ (1.82)	4.96^a MN^ (0.17)
	50	6.41^c I^ (1.43)	7.41^bc H^ (1.29)	8.46^ab IJ^ (1.01)	8.77^ab GH^ (1.26)	8.82^ab FGH^ (1.28)	10.05^a GHI^ (0.45)	3.46^b KLM^ (0.51)	4.44^b M^ (1.32)	4.46^b MN^ (1.85)	5.60^a KL^ (0.61)	5.67^a M^ (1.21)	6.07^a MN^ (1.62)
	55	7.73^c I^ (1.58)	8.73^bc H^ (1.73)	9.46^bc IJ^ (1.72)	9.82^b GH^ (0.28)	10.10^b FG^ (1.32)	11.71^a GH^ (1.49)	4.21^b KL^ (1.08)	4.83^b M^ (1.64)	4.96^b MN^ (0.11)	6.72^a KL^ (0.12)	7.15^a M^ (0.60)	7.19^a MN^ (1.51)
	60	9.05^c I^ (1.41)	10.05^bc H^ (0.86)	10.45^bc I^ (0.26)	10.81^bc G^ (0.17)	11.42^b F^ (1.19)	13.36^a G^ (1.23)	4.95^b K^ (1.43)	5.20^b M^ (1.30)	5.48^b M^ (1.28)	7.84^a K^ (0.84)	8.31^a M^ (1.35)	8.64^a M^ (1.11)
**Toxin + *T. ammi* leaves extract**	25	28.81^c H^ (1.98)	33.64^c G^ (1.54)	36.25^bc H^ (1.1)	43.17^bc F^ (2.03)	52.63^ab E^ (2.96)	62.16^a F^ (1.21)	34.60^e J^ (1.72)	39.06^de L^ (1.21)	44.27^cd L^ (0.70)	47.99^bc J^ (0.19)	51.71^ab L^ (1.52)	55.80^a L^ (1.08)
	30	34.17^c GH^ (1.84)	37.51^c G^ (1.65)	38.34^bc H^ (1.27)	43.91^bc F^ (1.46)	55.02^ab E^ (1.08)	63.35^a F^ (0.89)	36.09^d IJ^ (1.87)	40.80^cd L^ (1.61)	46.50^bc KL^ (1.17)	50.22^b IJ^ (0.85)	53.45^ab KL^ (0.59)	57.66^a KL^ (0.15)
	35	38.34^c FG^ (1.56)	41.97^c FG^ (1.87)	42.50^bc GH^ (1.18)	47.48^bc EF^ (1.49)	58.29^ab E^ (1.83)	66.03^a F^ (1.17)	37.57^d HIJ^ (1.65)	43.03^cd KL^(1.98)	48.73^bc JKL^(1.06)	51.71^b HIJ^ (0.97)	54.93^ab JKL^ (1.17)	59.15^a JKL^ (1.39)
	40	45.53^c EF^ (1.32)	49.42^c EF^ (1.21)	49.35^c FG^ (1.57)	54.63^bc DE^ (1.62)	65.74^ab D^ (1.74)	73.18^a E^ (1.87)	39.06^d G-J^ (1.97)	45.26^cd JK^ (1.53)	50.97^bc IJK^ (1.21)	53.20^b G-J^ (0.81)	56.42^ab IJK^ (0.43)	60.64^a IJK^ (1.17)
	45	47.02^c EF^ (1.70)	52.99^c DE^ (1.34)	52.63^c F^ (1.56)	55.82^bc D^ (1.39)	66.33^ab D^ (1.32)	76.75^a DE^ (1.25)	40.55^d F-I^ (1.78)	47.49^cd IJ^ (1.42)	53.20^bc HIJ^ (1.15)	54.69^b F-I^ (0.82)	57.91^ab HIJ^ (0.55)	62.13^a HIJ^ (1.19)
	50	47.22^c EF^ (1.65)	52.18^c DE^ (1.42)	53.22^c EF^ (1.19)	56.71^bc D^ (0.96)	66.63^b D^ (0.73)	77.94^a D^ (1.59)	42.04^d FGH^(1.31)	49.73^c HI^ (1.87)	55.43^bc GHI^ (1.20)	56.17^bc FGH^ (1.28)	59.40^ab GHI^ (1.32)	63.62^a GHI^ (1.36)
	55	49.70^c DE^ (1.29)	55.08^c CDE^ (1.37)	56.20^bc DEF^(1.17)	59.99^bc D^ (1.16)	67.25^ab C^ (1.1)	78.24^a D^ (1.04)	43.53^d FG^ (1.56)	51.96^c GH^ (1.42)	57.66^bc GH^ (0.86)	57.66^bc FG^ (1.14)	60.89^ab GH^ (1.07)	65.10^a GH^ (1.28)
	60	51.79^c DE^ (1.37)	56.56^c CDE^ (1.45)	56.50^c DEF^ (1.62)	60.58^bc D^ (1.79)	68.51^ab CD^ (2.13)	79.43^a CD^ (1.96)	45.01^d F^ (1.78)	54.19^c G^ (1.12)	59.90^abc G^ (1.58)	59.15^bc F^ (1.29)	62.38^ab G^ (1.19)	66.59^a G^ (1.09)
**Toxin + *T. ammi* leaves extract**	25	52.39^d DE^ (1.21)	55.39^d CDE^ (1.08)	61.86^cd CDE^ (1.19)	68.48^bc C^ (1.23)	73.21^ab BC^ (1.37)	79.77^a CD^ (1.28)	69.26^b E^ (1.56)	72.24^ab F^ (1.67)	75.22^ab F^ (0.58)	76.70^a E^ (1.29)	78.19^a F^ (1.44)	78.94^a F^ (0.96)
	30	57.75^d CD^ (1.38)	59.26^cd CD^ (1.55)	63.94^cd CD^ (1.73)	69.22^bc C^ (1.90)	75.59^ab B^ (1.12)	80.97^a CD^ (1.04)	70.75^c DE^ (1.45)	73.98^bc F^ (1.86)	77.45^abc EF^(1.24)	78.94^ab DE^ (1.31)	79.93^ab EF^ (1.20)	81.17^a EF^ (1.17)
	35	61.92^d BC^ (1.67)	63.72^d BC^ (1.23)	68.11^cd BC^ (1.47)	72.79^bc BC^ (1.26)	78.86^ab B^ (1.16)	83.65^a C^ (1.02)	72.24^b CDE^ (1.54)	76.21^ab EF^ (1.38)	79.68^b DEF^ (1.63)	80.42^a CDE^ (1.51)	81.42^a DEF^ (1.65)	82.66^a DE^ (1.13)
	40	69.11^d AB^ (1.56)	69.98^d AB^ (1.87)	74.96^cd AB^ (1.23)	79.94^bc AB^ (1.22)	86.31^ab A^ (1.06)	88.41^a B^ (0.89)	73.73^b B-E^ (1.09)	78.44^ab DE^ (1.34)	81.88^a CDE^ (1.78)	81.91^a B-E^ (1.81)	82.90^a CDE^ (1.35)	84.14^a CDE^ (1.79)
	45	70.60^e AB^ (0.61)	74.44^de A^ (1.78)	78.23^cd A^ (0.99)	81.13^bc A^ (1.12)	86.90^ab A^ (1.34)	91.98^a AB^ (1.42)	75.22^b A-D^ (1.21)	80.67^ab CD^ (1.85)	84.14^a BCD^ (1.56)	83.40^a A-D^ (1.07)	84.39^a BCD^ (1.54)	85.63^a BCD^ (0.66)
	50	70.44^d AB^ (1.42)	73.65^d A^ (1.87)	78.83^c A^ (1.16)	81.43^c A^ (1.22)	87.20^b A^ (1.33)	93.17^a A^ (1.07)	76.70^b ABC^(1.82)	82.90^ab BC^ (1.36)	86.38^a ABC^ (1.13)	84.89^a ABC^ (1.07)	85.88^a ABC^ (0.84)	87.12^a ABC^ (1.18)
	55	72.09^d A^ (1.66)	75.78^d A^ (1.32)	81.81^c A^ (0.85)	84.70^bc A^ (1.87)	88.58^ab A^ (1.34)	93.47^a A^ (1.19)	78.19^b AB^ (1.36)	85.14^ab AB^ (1.24)	88.61^a AB^ (1.02)	86.38^a AB^ (0.87)	87.37^a AB^ (1.72)	88.61^a AB^ (1.92)
	60	72.69^e A^ (1.09)	77.27^de A^ (1.75)	82.11^cd A^ (1.65)	85.30^bc A^ (1.82)	89.09^b A^ (1.73)	94.66^a A^ (1.22)	79.68^b A^ (1.52)	87.37^a A^ (1.31)	90.84^a A^ (1.82)	87.86^a A^ (1.30)	88.86^a A^ (1.67)	90.10^a A^ (1.17)

Values are mean of three replicates.

Small alphabetic letters with different values indicate significant differences (p < 0.05) in toxin reduction at different temperature and incubation periods in each row.

Capital alphabetic letters with different values indicate significant differences (p < 0.05) among controls and tested plant extracts at different temperature and incubation period in each column. Standard deviation values are shown in parenthesis.

It is evidenced from the results that percentage detoxification of AFB1 and AFB2 by *T. ammi* extracts was progressively increased with the consequent increase in temperature from 30 to 55°C. The highest inactivation was observed at 60°C. At this temperature, respective control (water) showed 10.4 and 8.6% detoxification of AFB1 and AFB2 after 72 h of incubation. However, toxin treated with *T. ammi* leaves and seeds extract showed 79.46 and 94.7% detoxification of aflatoxin B1 while detoxification of aflatoxin B2 was 66.6 and 90.1% respectively, under same conditions. This detoxification may be due to synergistic effect of heat and moisture (Table 1).

In the present investigation, it was found that *T. ammi* seeds extract was effective in detoxifying aflatoxin B1 and B2 at all tested temperatures and incubation periods. However, for further studies, 30°C was selected as it is more or less near to room temperature and moreover was found close to be the existing temperature of storehouses in Punjab especially in summer. Therefore, by selecting this temperature cost of maintaining temperature in storehouses can be greatly reduced.

### Effect of pH on toxin detoxification by *T. ammi* extracts (In vitro)

**Figure 1:**
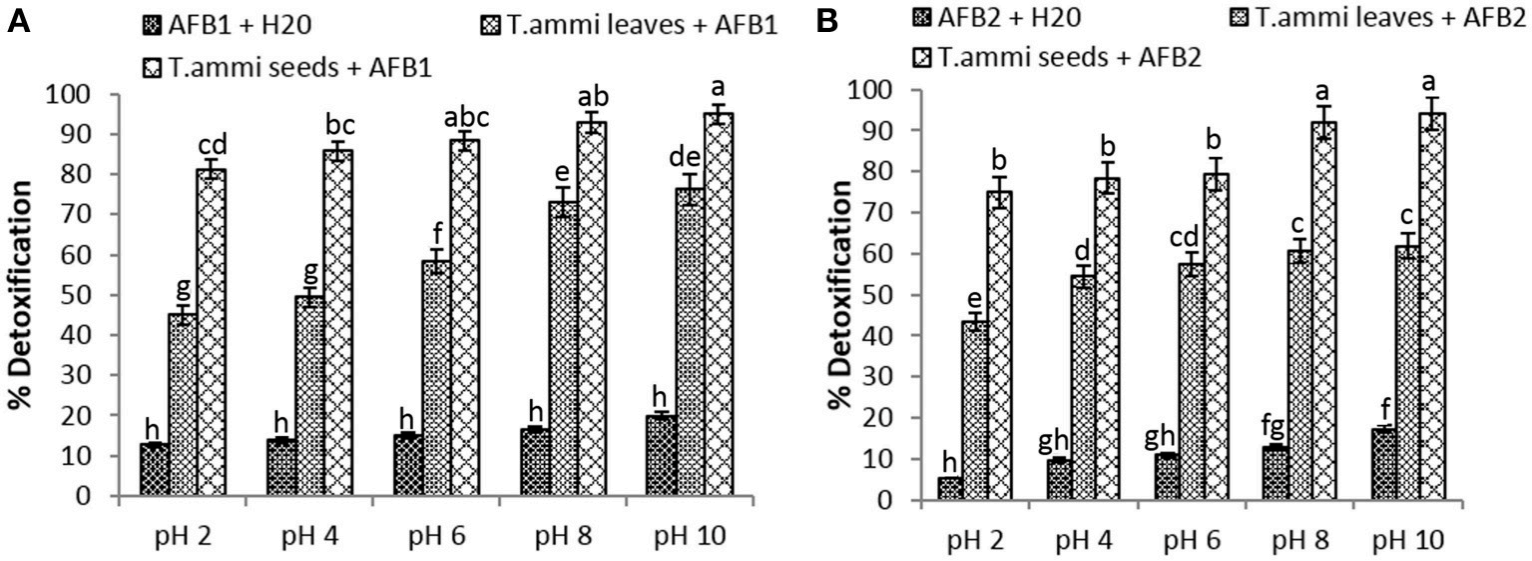
**Effect of pH on detoxification of aflatoxin by aqueous extracts of *T. ammi***. Whereas **(A)** AFB1; **(B)** AFB2.

The comparative assessment of *T. ammi* leaves and seeds extract to detoxify AFB1 and AFB2 at different pH values revealed that least significant detoxification occurred at pH 2. Results indicated that at pH 2, percentage reduction of AFB1 and AFB2 was 81.2 and 74.9% respectively in samples treated with *T. ammi* seeds extract after 72 h of incubation at 30°C.The efficacy of *T. ammi* seeds extract to detoxify AFB1 and AFB2 was significantly (*P* < 0.05) increased with increase in pH from 4 to 10 (Figure 1). Distilled water with pH adjusted to 2, 4, 6, 8, and 10 was used as a control. Control data showed that at pH 10, 19.94% of AFB1 and 17.45% of AFB2 was degraded after 72 h of incubation at 30°C while 15.88 and 13.05% degradation of AFB1 and AFB2 was observed at pH 8 under same conditions. The percentage of degradation decreases as the pH decreases to neutral or acidic range.

Maximum degradation of AFB1 and AFB2 was observed at pH 10 after treatment with *T. ammi* seeds extract i.e., 94.9 and 94.1% as compared to *T. ammi* leaves extract with degradation percentage of 76.2 and 61.9% respectively.

However, at high basic pH conditions aflatoxins are known to become unstable and sensitive, therefore to avoid this in further experimentations pH 8 was selected which is 100 times less alkaline than pH 10. It was also evidenced from the results that *T. ammi* seeds extract significantly (*P* < 0.05) detoxified AFB1 and AFB2 at slightly alkaline pH 8 and their results were closely comparable with the results obtained at pH 10 (Figure 1).

### Effect of boiling on toxin detoxification properties of *T. ammi* extracts

The aflatoxins detoxification efficacy of *T. ammi* leaves and seeds extract was significantly (*P* < 0.05) decreased upon boiling at 100°C for 10 min. The pH of treated (boiled) and untreated (Unboiled) extracts was adjusted to 8. Unboiled *T. ammi* leaves extract showed 73.0 and 60.6% detoxification of AFB1 and AFB2 as compared to boiled extracts i.e., 54.4 and 45.7% respectively. Similarly 92.8 and 91.9% detoxification of AFB1 and AFB2 was recorded after treatment with unboiled *T. ammi* seeds extract in comparison with boiled seeds extract with 69.9 and 74.4% detoxification. Results indicated that upon boiling, AFB1 detoxifying activity of *T. ammi* seeds and leaves extract were decreased up to 23 and 18% respectively. While in case of AFB2, 15 and 17.4% decrease in detoxification was recorded after treatment with boiled extracts of *T. ammi* leaves and seeds respectively. So, these results clearly depicted that unboiled plants extracts are more efficient in degrading aflatoxins as compared to boiled extracts.

### *In vivo* detoxification of aflatoxins in maize samples

*In vivo* analysis followed a similar trend as that was recorded in *in vitro* studies. These studies were carried out under conditions optimized in previous *in vitro* assays i.e., pH 8, temperature 30°C and incubation time 72 h. Data obtained from *in vivo* studies showed that maximum detoxification of AFB1 and AFB2 in spiked maize samples was carried out by *T. ammi* seeds extract after 72 h of incubation i.e., 89.6 and 86.5% respectively. As compared to *T. ammi* seeds extract, in *T. ammi* leaves extract 68.8 and 53.7% detoxification of AFB1 and AFB2 was observed in spiked samples (Table 2).

**Table 2:** ***In vivo* detoxification of AFB1 and AFB2 by aqueous extracts of *T. ammi***.

	**Toxin recovery (μg L^−1^)**	
	**AFB1**	**AFB2**
**CONTROL**		
Unspiked maize Unspiked maize + *T. ammi* leaf extract(s) Unspiked maize + *T. ammi* branch extract Spiked maize with AFB1(100ng/ml) and AFB2 (50 ng/ml)	0.49^a^ 0.00^a^ 0.00^a^ 97.30^i^	0.33^a^ 0.00^a^ 0.00^a^ 47.65^n^
**TREATMENTS**		
Spiked maize with toxin + *T. ammi* leaf extract (s) Detoxification (%) Spiked maize with toxin + *T. ammi* seeds extract Detoxification (%)	31.2^d^ 68.8 10.4^ab^ 89.6	23.1^ef^ 53.7 6.8^bc^ 86.5

Values are mean of three replicates.

Data were analyzed by analysis of Variance (ANOVA).

Values with different letters show significant difference (P = 0.05) as determined by Tukey's Multiple Range test.

**Figure 2:**
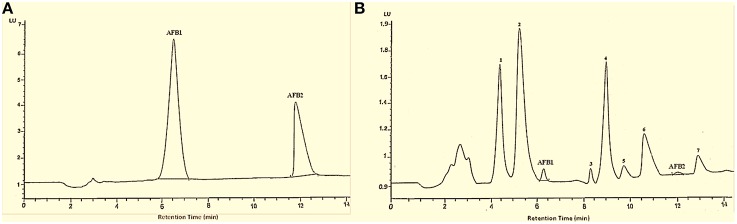
**HPLC chromatogram of AFB1 and AFB2**. Whereas **(A)** untreated toxins; **(B)** toxin treated with *T. ammi* seeds extract at 30°C and pH 8.

HPLC chromatograms confirmed that after *T. ammi* seeds extract treatment, trace amount of aflatoxin was present along with other peaks whose footprints were not found in chromatogram of parent compounds which may be attributed to toxins degradation products (Figure 2).

### Structural characterization of AFB1, AFB2, and their degradation products

Both aflatoxin B1 and B2 exhibited good ESI ionization efficiency in the positive ion mode with molecular base ion at m/z 313.17 and m/z 315.17 for protonated adduct [M+ H]^+^ while m/z 335 and m/z 337 for sodium adduct [M+ Na]^+^ respectively. Identity of parent compound was validated by its fragmentation into daughter ions. The protonated molecule was chosen as the precursor ion for aflatoxins in the product ion scan mode because the sodium adduct did not exhibit specific fragmentation for any compound.

### MS/MS analysis of AFB1 and AFB2

**Figure 3:**
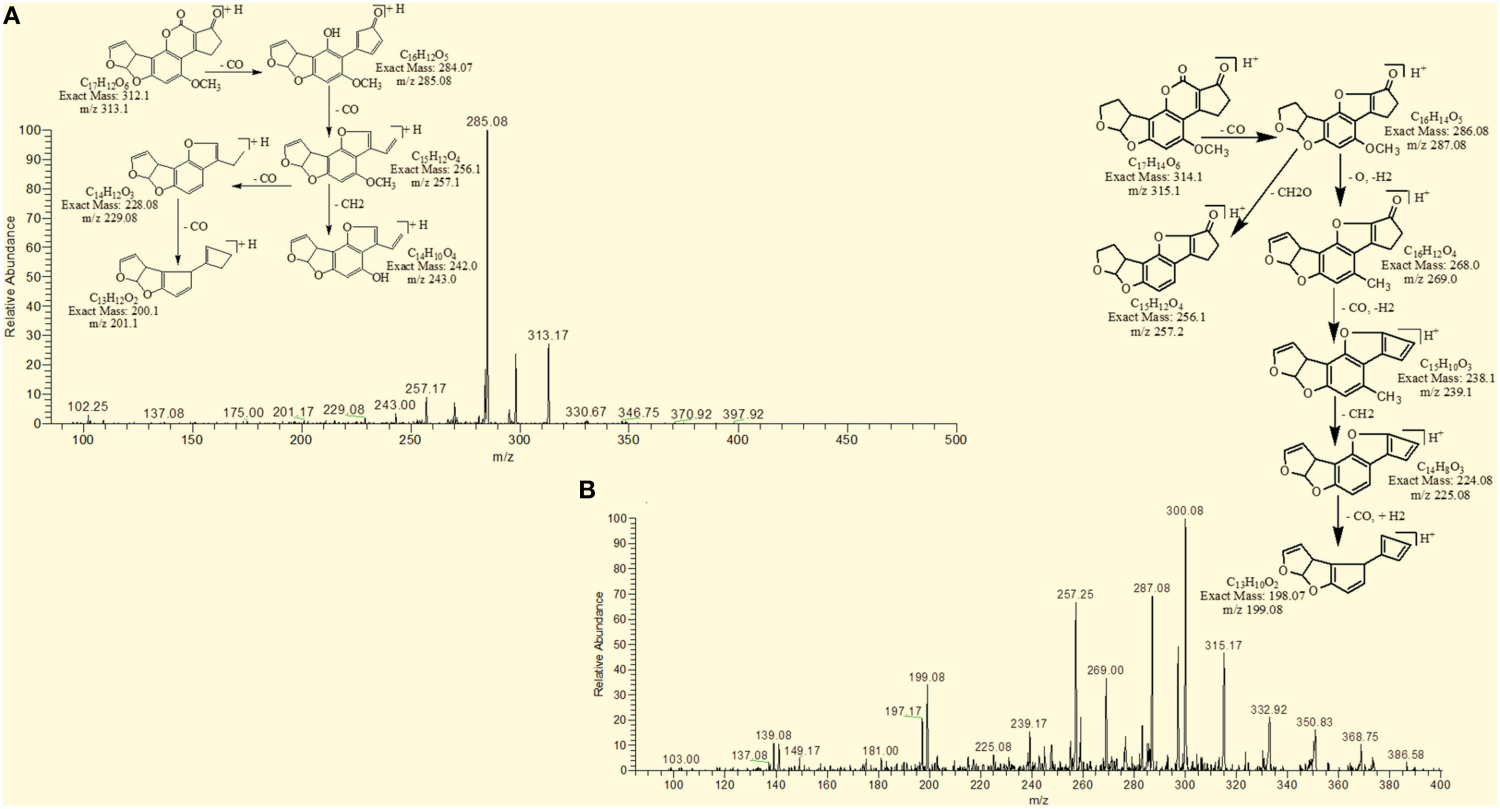
**MS/MS Spectra and fragmentation pathway. (A)** AFB1 and **(B)** AFB2.

MS/MS spectrum of AFB1 showed that continuous loss of carbon monoxide (CO) was the main fragmentation pathway. Methyl and methanol losses occurred on methoxy group located on side chain of benzene. The double bond equivalence (DBE) of AFB1 was 12 (Figure 3A). However, MS/MS fragmentation pathway of AFB2 revealed that daughter ions were formed by loss of carbon monoxide, oxygen, hydrogen and methyl group (Figure 3B). The DBE of AFB2 was 11. The identification of degradation products were based on accurate mass measurement of ions and similar fragmentation pathways with AFB1 and AFB2.

**Figure 4:**
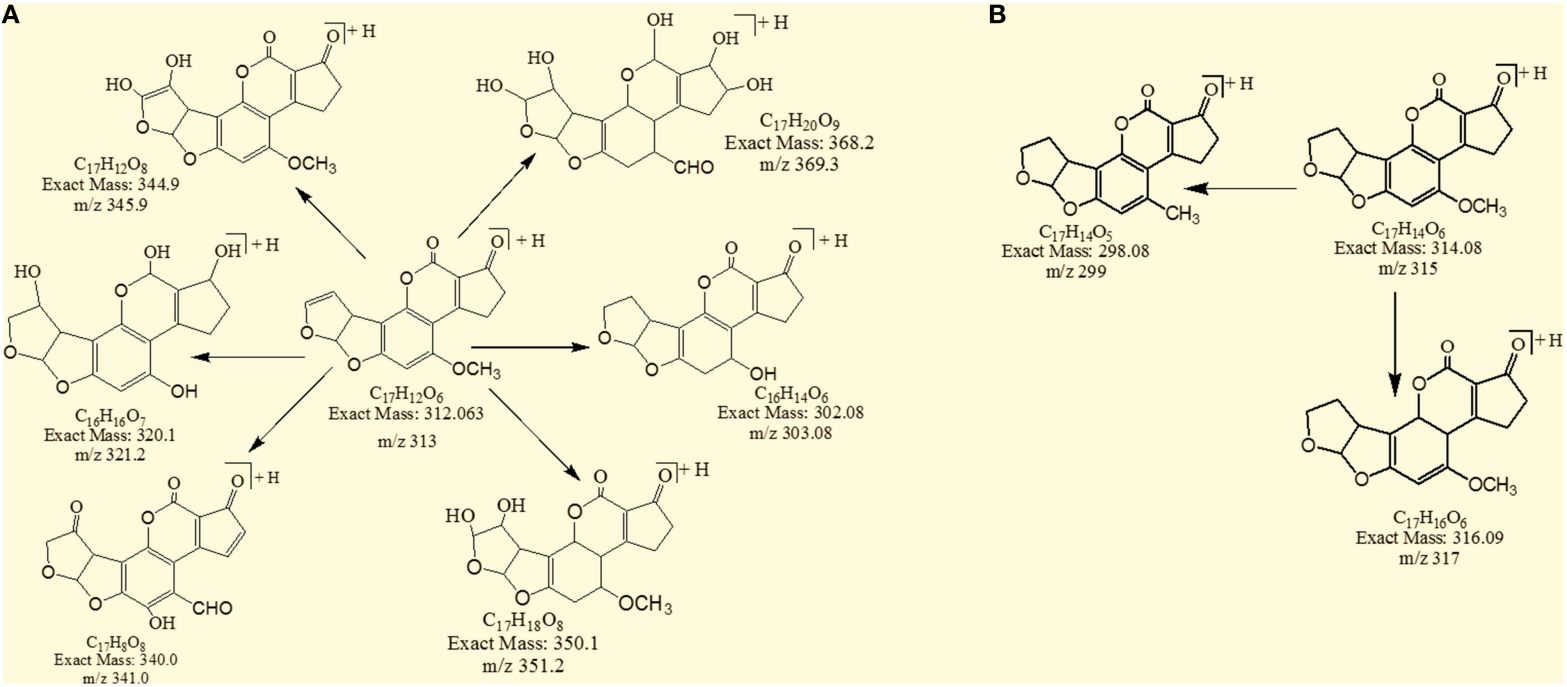
**Possible degraded products of (A) AFB1 and (B) AFB2 after treatment with *T. ammi* seeds extracts at 30°C and pH 8**.

Results showed that in most of the degraded products obtained after treatment with *T. ammi* seeds extract additional reactions occurred which leads to the loss of double bond in terminal furan ring responsible for toxicity. Structural formulas of possible hypothesized degraded products of AFB1 and AFB2 are shown in Figures 4A,B.

### MS/MS analysis for confirmation of degraded products of AFB1

**Figure 5:**
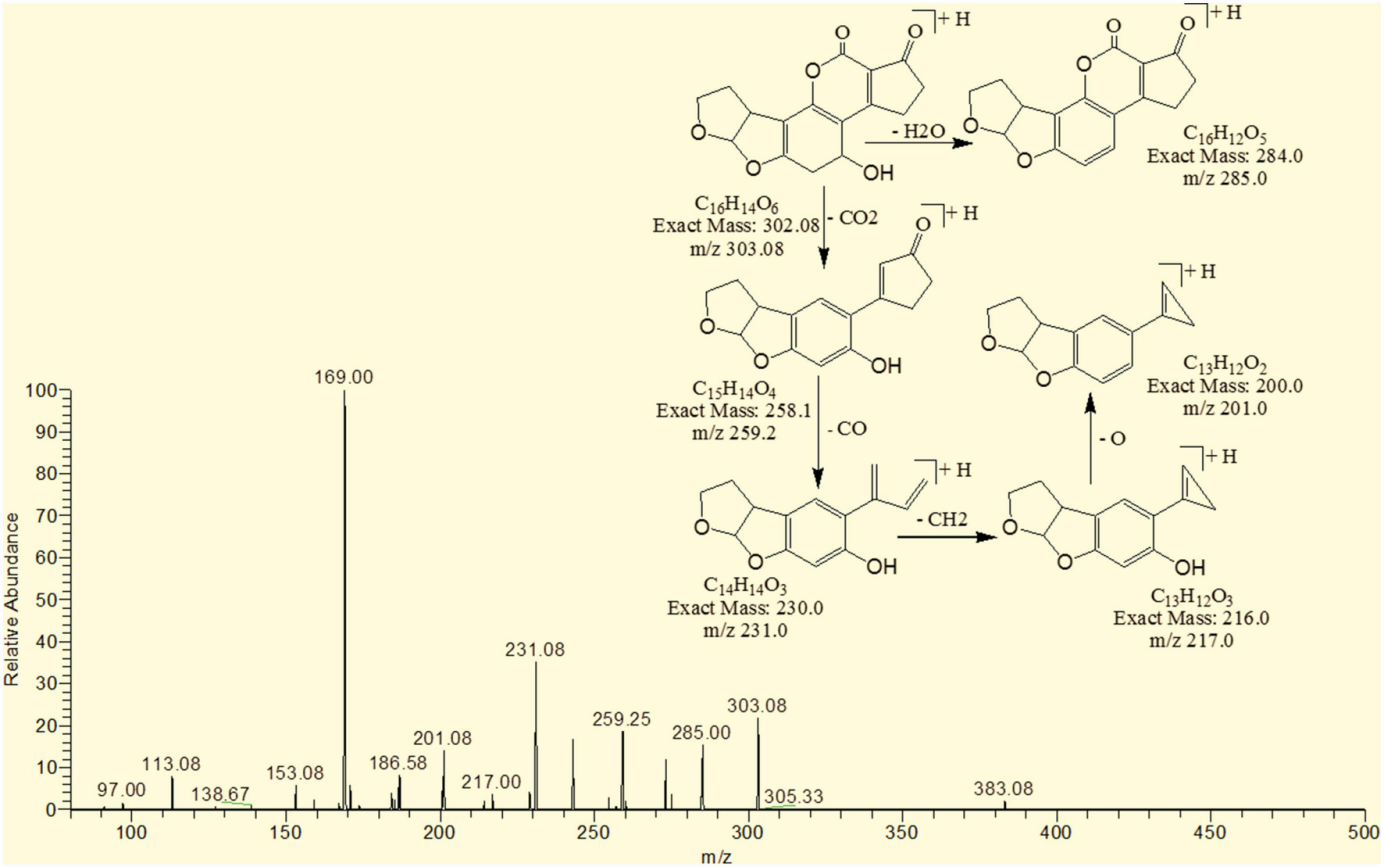
**MS/MS spectra and fragmentation pathway of degradation product with 303.08 m/z**.

The degradation product at m/z 303.08 corresponded to molecular formulae C_16_H_14_O_6_ was formed due to the elimination of CH_2_ and addition of hydrogen atoms. The DBE of C_16_H_14_O_6_ was less than AFB1 i.e., 10. Loss of H_2_O, CO_2_, CO and O was the main fragmentation pathway (Figure 5).

**Figure 6:**
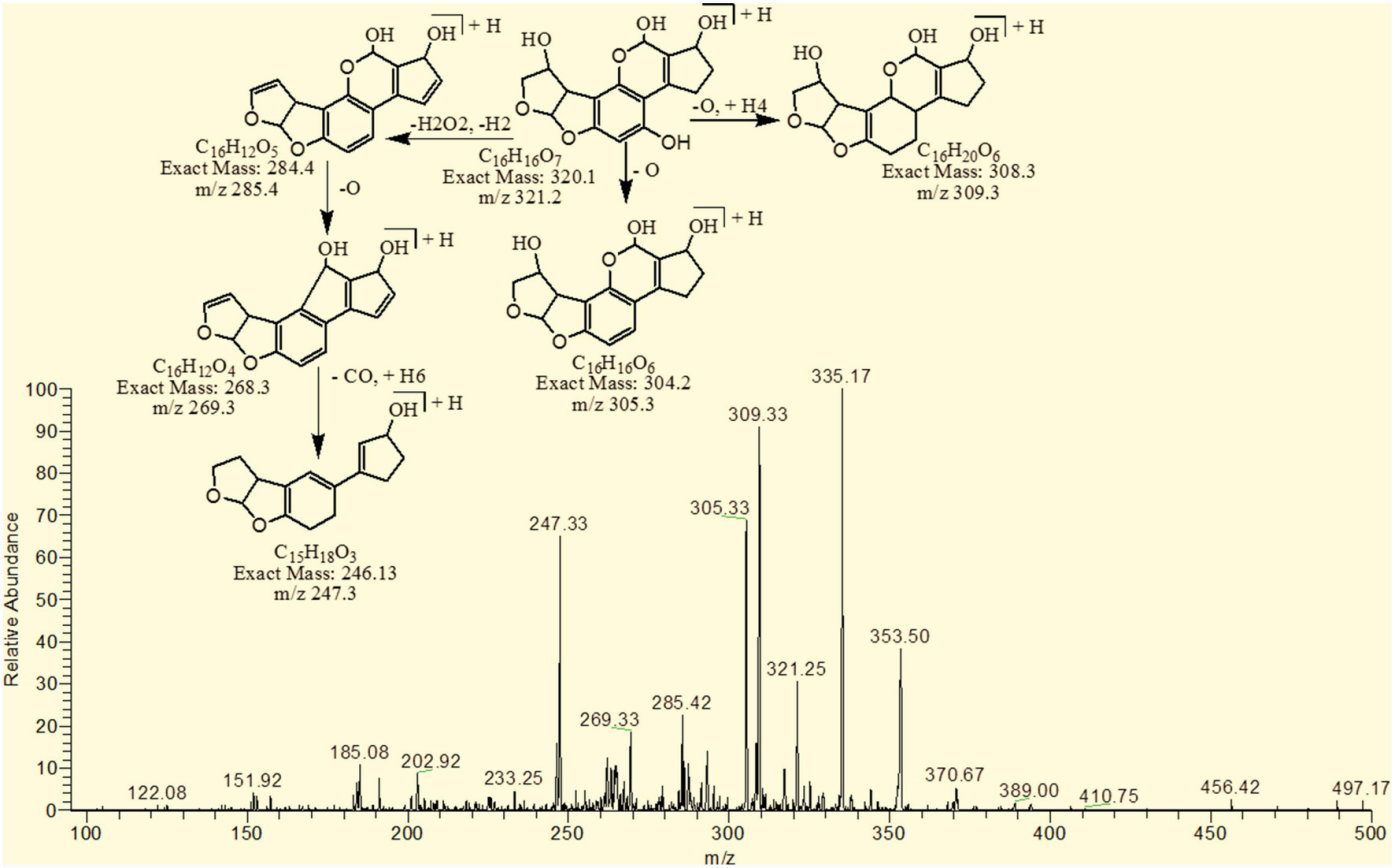
**MS/MS spectra and fragmentation pathway of degradation product with 321.25 m/z**.

The degradation product C_16_H_16_O_7_ (with 321.25 m/z) was formed due to the addition of hydroxyl group on the terminal furan ring and replacement of methoxy group on the side chain of benzene ring with hydroxyl group. The DBE of C_16_H_16_O_7_ content was lower than AFB1 i.e., 9. Fragments demonstrated that the loss of oxygen and carbon monoxide was the main fragmentation pathway which was different from that of AFB1 (Figure 6).

**Figure 7:**
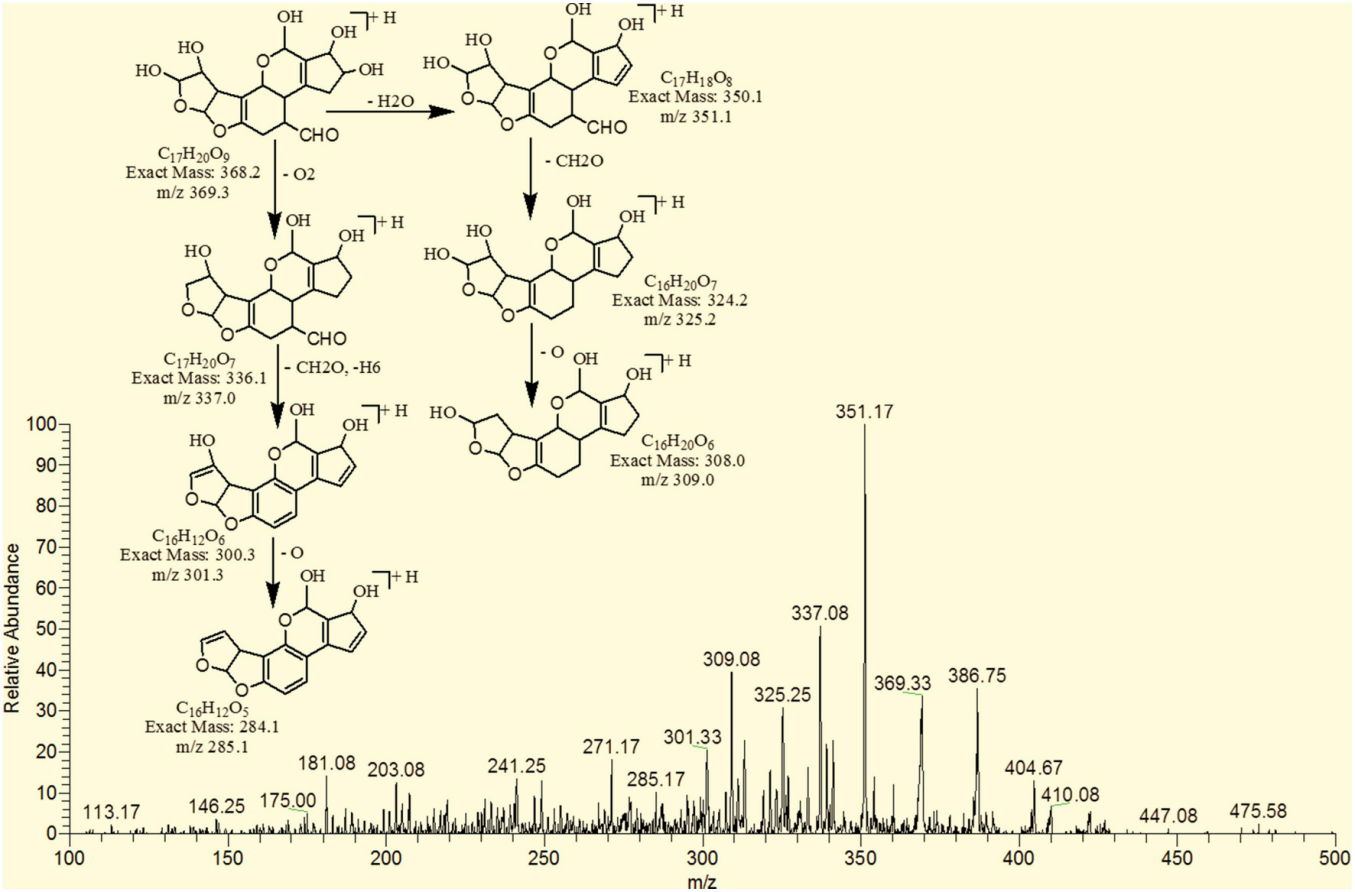
**MS/MS spectra and fragmentation pathway of degradation product with 369.33 m/z**.

The degradation product C_17_H_20_O_9_ (with 369.33 m/z) had more H_8_O_3_ molecules than AFB1. The DBE of C_17_H_20_O_9_ was 7, which was lower than AFB1 implying that additional reactions occurred on the furan rings. Fragmentation pathway was different from that of AFB1. The precursor ion yielded a series of product ions which were represented by 351.17 [M-H_2_O]^+^, 337.08 [M-O_2_]^+^, 325.25 [M-CO_2_]^+^, 309.08 [M-CO_3_]^+^, 301.33 [M-CH_8_O_3_]^+^ and 285.17 [M-CH_8_O_4_]^+^ (Figure 7).

**Figure 8:**
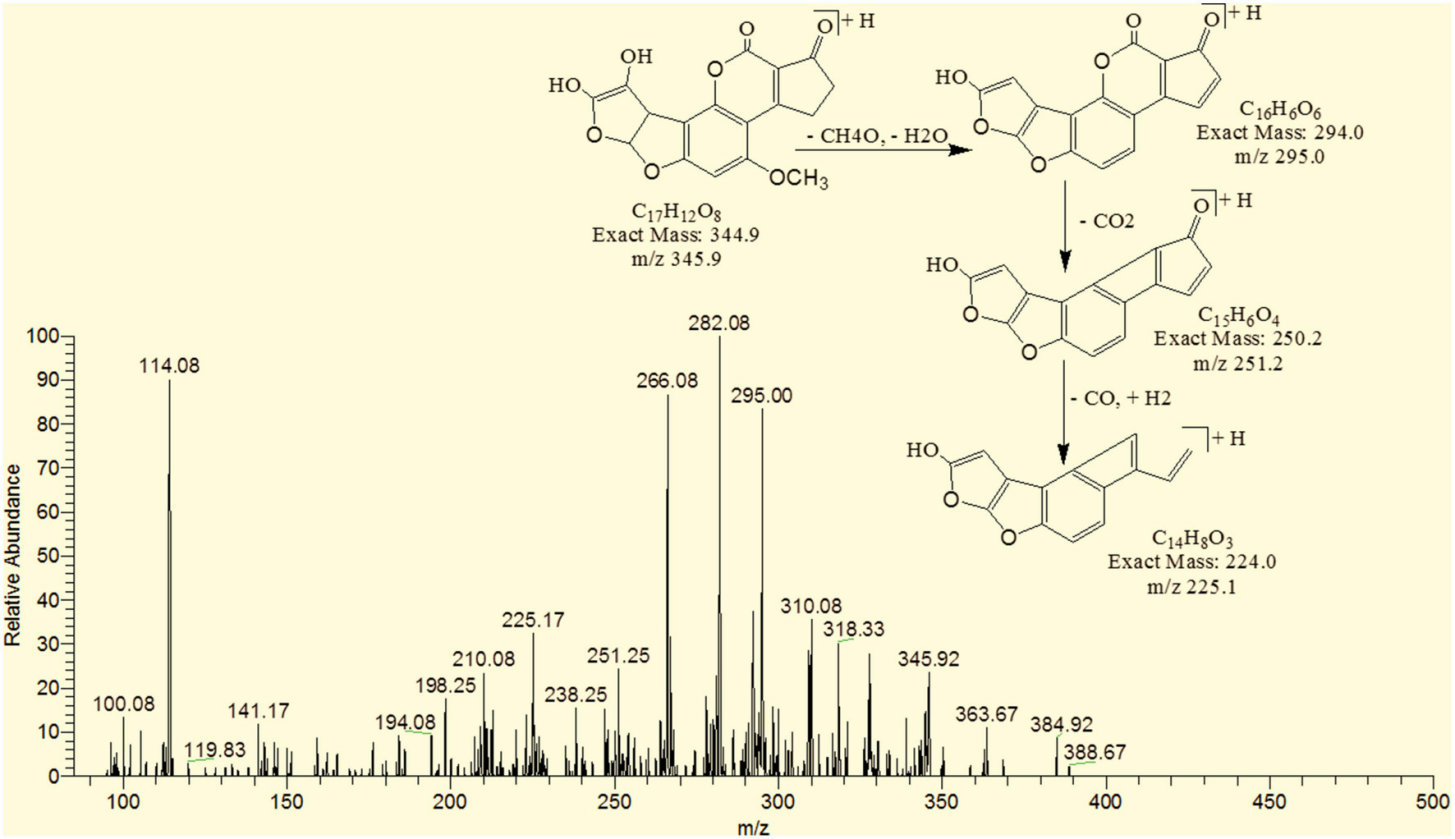
**MS/MS spectra and fragmentation pathway of degradation product with 345.92 m/z**.

The degradation product 345.92 corresponded to molecular formula C_17_H_12_O_8_ was formed due to the addition of hydroxyl groups on the terminal furan ring. The DBE of C_17_H_12_O_8_ was same as that of AFB1 i.e., 12. The fragments of C_17_H_12_O_8_ showed losses of CH_4_O, H_2_O, CO_2_ and CO. More details on the fragmentation pathway are shown in Figure 8.

**Figure 9:**
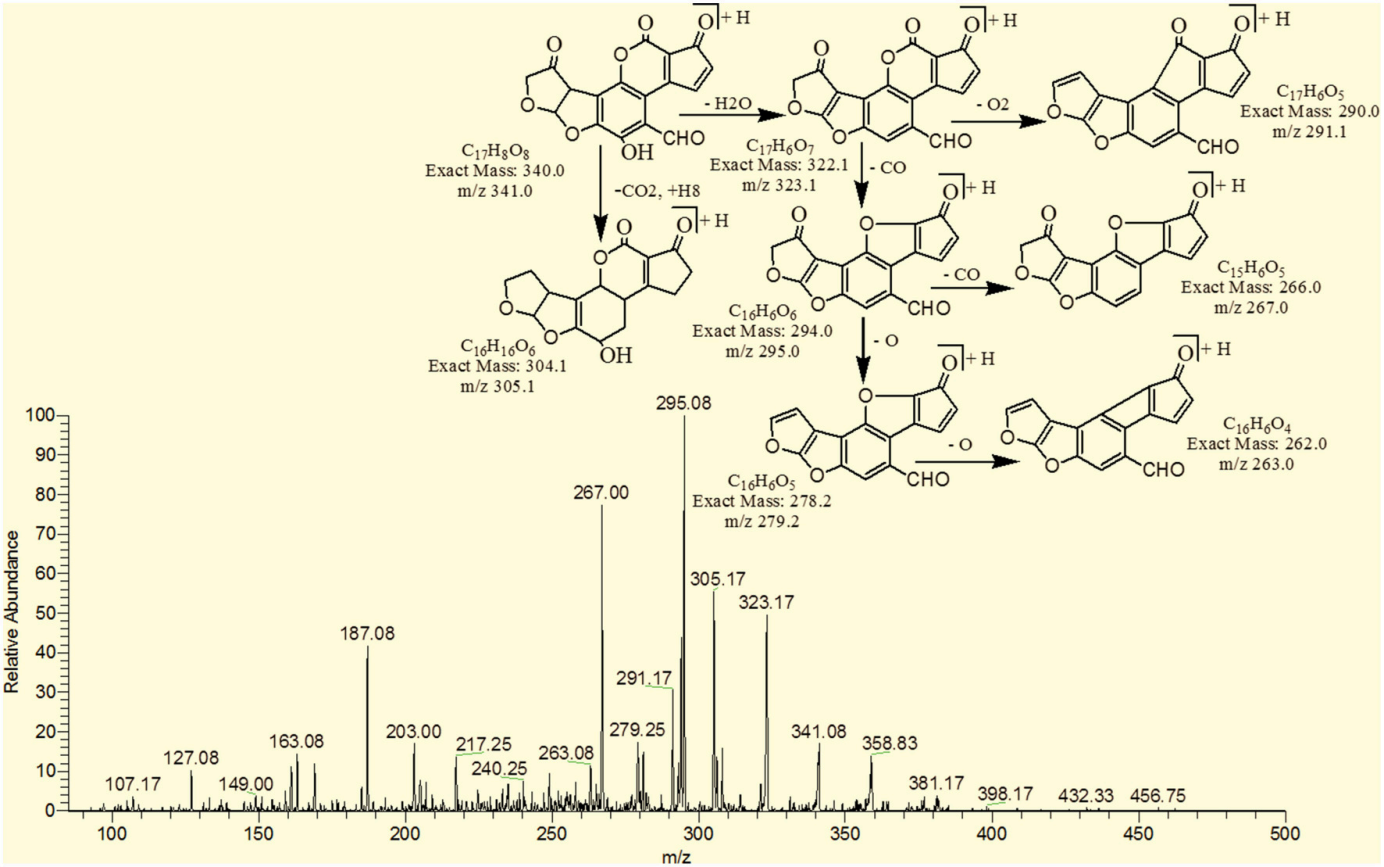
**MS/MS spectra and fragmentation pathway of degradation product with 341.08 m/z**.

The degradation product C_17_H_8_O_8_ (with 341.08 m/z) had two more oxygen and four less hydrogen atoms. Addition of oxygen occurred on the terminal furan ring and benzene ring. The DBE of C_17_H_8_O_8_ was one more than AFB1 i.e., 13. Series of product ions formed by the precursor ion were represented by 323.17 [M-H_2_O]^+^, 295.08 [M-CH_2_O_2_]^+^, 291.17 [M-H_2_O_3_]^+^, 279.25 [M-CH_2_O_3_]^+^, 267.0 [M-C_2_H_2_O_3_]^+^, 263.08 [M-CH_2_O_4_]^+^ (Figure 9).

**Figure 10:**
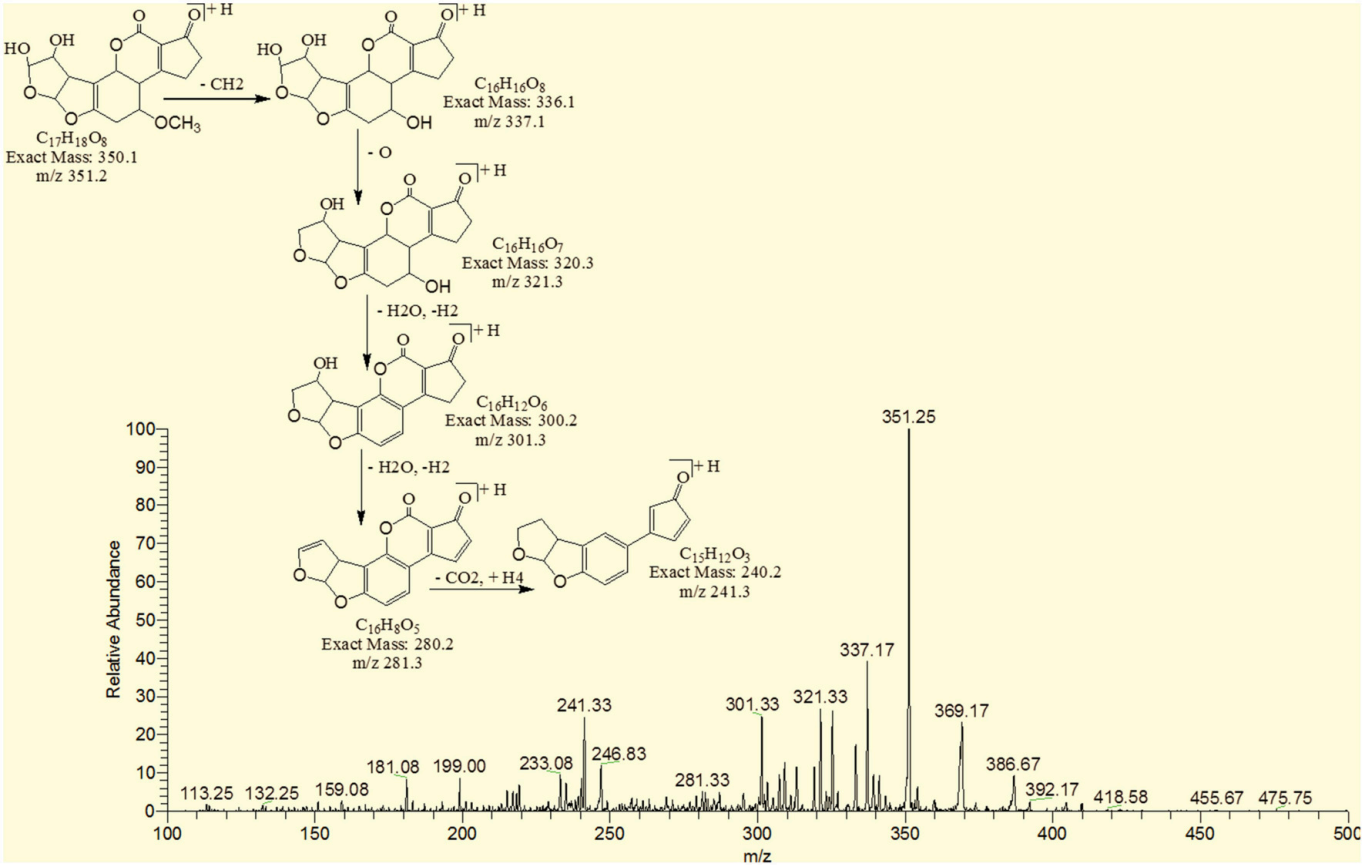
**MS/MS spectra and fragmentation pathway of degradation product with 351.25 m/z**.

The degradation product C_17_H_18_O_8_ (with 351.25 m/z) was formed by addition of two hydroxyl groups on the double bond of terminal furan ring. The DBE of C_17_H_18_O_8_ was less than AFB1 i.e., 9 with different fragmentation pathway from that of AFB1. Product ions formed from parent ion C_17_H_18_O_8_ was represented by 337.17 [M-CH_2_]^+^, 321.33[M-CH_2_O]^+^, 301.33[M-CH_6_O_2_]^+^, 281.33[M-CH_10_O_3_]^+^ and 241.33[M-C_2_H_6_O_5_]^+^ (Figure 10).

### MS/MS analysis for confirmation of degraded products of AFB2

**Figure 11:**
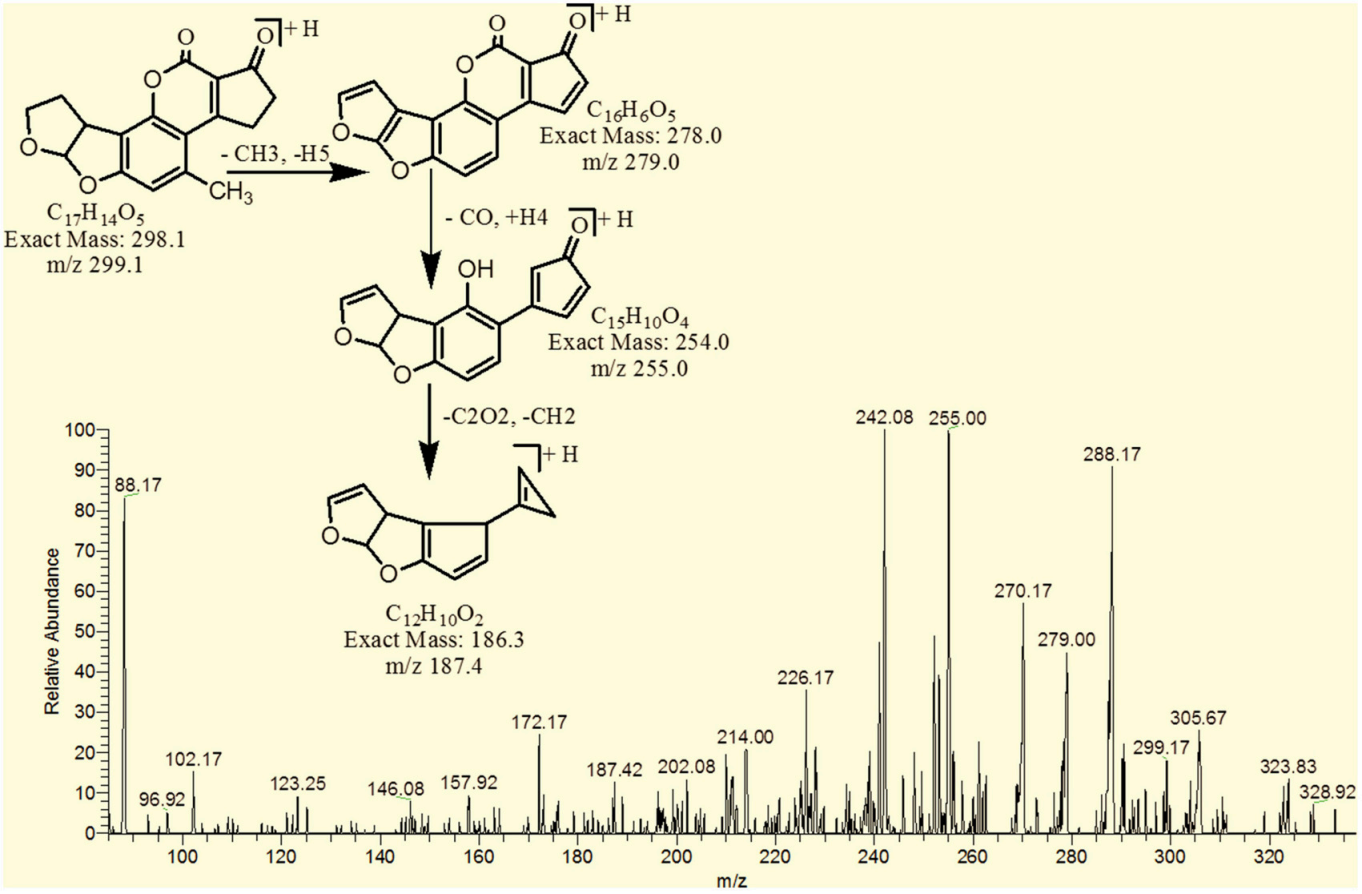
**MS/MS spectra and fragmentation pathway of degradation product with 299.17 m/z**.

The degradation product C_17_H_14_O_5_ (with m/z 299.17) was formed by the loss of oxygen atom from the side chain of benzene ring. The DBE of C_17_H_14_O_5_ was same as that of AFB2. Fragments of C_17_H_14_O_5_ showed losses of CH_3_, C_2_O_2_ and CO. More detail on fragmentation pathway are shown in Figure 11.

**Figure 12:**
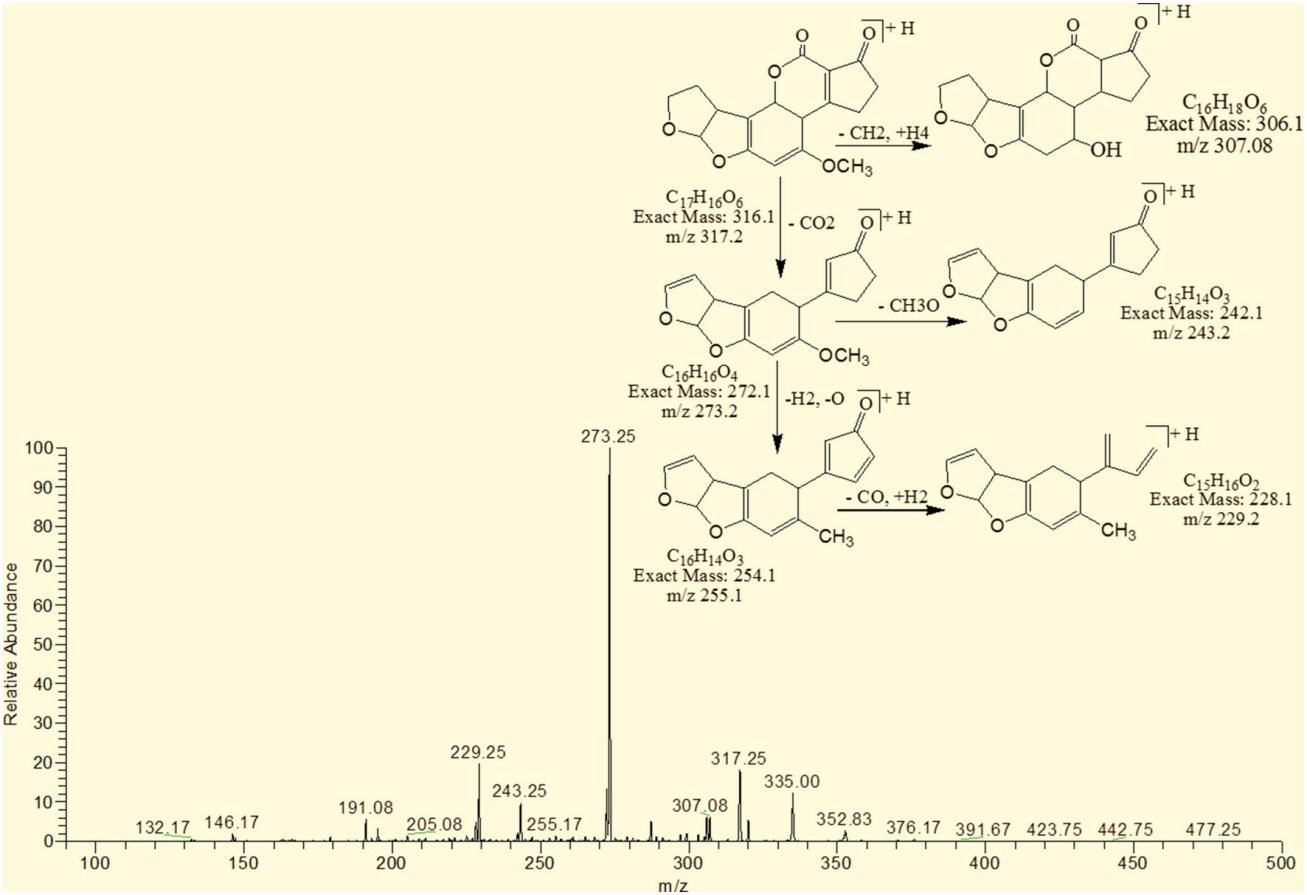
**MS/MS spectra and fragmentation pathway of degradation product with 317.25 m/z**.

However, the degradation product C_17_H_16_O_6_ at m/z 317.25 had two more hydrogen atoms and one less DBE than AFB2. Loss of CO_2_, CO, CH_3_O and O was the main fragmentation pathway (Figure 12).

### Assessment of biological toxicity of degraded products

Biological toxicity of degraded toxin products were tested using brine shrimps (*Artemia salina*) bioassay. The brine shrimps assay actually proved to be a convenient system for monitoring biological activity (Hartl and Humpf, 2000). In this study, the degraded toxin products were incubated with brine shrimp larvae at 26°C for 24–96 h. The percentage of mortality was compared with that of control (Table 3).

**Table 3:** **Percent mortality of brine shrimps (*Artemia salina*) larvae at 26°C after treatment with toxin (AFB1 and AFB2) detoxified with *T. ammi* seeds extract at various incubation periods**.

**Treatments**	**Incubation period (h)**	**No. of living shrimps**	**No. of dead shrimps**	**% Mortality**
**CONTROL**				
Sea water + shrimps	24 48 72 96	40 40 40 39	0 0 0 1	0 0 0 2.5
Methanol + shrimps	24 48 72 96	38 38 37 36	1 2 3 3	2.5 5 7.5 7.5
Untreated toxins + shrimps	24 48 72 96	7 5 4 3	33 35 36 37	83.0 86.7 89.2 91.7
**TREATMENT**				
Treated toxin with *T. ammi* seeds extract + shrimps	24 48 72 96	35 34 32 31	5 6 8 9	11.7 15.0 20.0 23.3

Sea water was taken as a control. Other Controls consist of Methanol and untreated toxin AFB1 (100 ng/ml) and AFB2 (50 ng/ml) dried and redissolved in sea water.

Values are mean of three replicates.

Data were analyzed by analysis of Variance (ANOVA).

Results indicated that only 11.7–23.3% mortality in brine shrimps larvae was observed after treatment with degraded toxin products. However, mortality level was 83.0–91.7% when larvae were incubated with untreated AFB1 (100 μg L^−1^) and AFB2 (50 μg L^−1^), under same conditions. Percentage of mortality was increased with increase in incubation period.

## Discussion

Various food and feed additives like phenolic compounds and plant extracts can be used to reduce toxic effects of mycotoxins (Nahm, 1995; Dvorska et al., 2007). According to the literature, essential oils and extracts of various spices and herbs like cinnamon, peppermint, basil, lemongrass may be recommended as a plant based safe food additive in protecting the food from deteriorating fungi as well as from aflatoxin contamination (Montes-Belmont and Carvajall, 1998; Burt, 2004; Yang et al., 2007). Similarly, there are several mycotoxins binding commercial products. Some of them are developed and approved in North America and Western Europe, such as Mycofix®and Mycosorb®. These binders are the combination of various things including herbal and yeast cell wall component extracts (Marroquin-Cardona et al., 2009). They have been used worldwide to neutralize or detoxify the mycotoxins in poultry, pig, ruminant feed as well as fish and shrimp diets. The aforementioned products are being extensively used in Pakistan.

In this present study, both *in vitro* and *in vivo* assays were performed with aqueous extracts of *T. ammi* leaves and seeds to check their aflatoxin B1 and B2 detoxification potential under optimized conditions of temperature, pH and incubation period. The results of *in vitro* assays showed that the percentage of detoxification by plants extracts increased with increase in temperature to 60°C but this detoxification could be due to synergistic action of heat and moisture (Basappa and Shantha, 1996; Rustom, 1997). Similarly, Hajare et al. (2005) worked on aflatoxin inactivation by using Ajwain seeds extract under optimized conditions. According to his findings, highest inactivation was observed at 60°C but further studies were conducted on 45°C to reduce the effect of heat and moisture on toxin inactivation.

The pH of reaction mixture plays an important role in the process of detoxification by using plant extracts. The maximum detoxification was observed at pH 10 followed by pH 8. The percentage of detoxification decreased as the pH changed to neutral or acidic range. Subsequently, Mendez-Albores et al. (2004) also found that aflatoxin florescence, attributed to the coumarin moiety, diminish or even disappear in alkaline treatment. In addition, the similar results were in accordance with the findings of Kannan and Velazhahan (2014) who explored the potential of *Barleria lupulina* leaf extract on detoxification of aflatoxins. Results of the present study showed that *in vivo* decontamination of maize samples followed a similar trend as that was recorded in *in vitro* studies. These studies were carried out under conditions optimized in previous *in vitro* assays i.e., pH 8, temperature 30°C and incubation time of 72 h.

Furthermore, *T. ammi* plant extracts showed varied degree of reduction in aflatoxin B1 and B2 detoxification upon boiling. Similar findings were recorded in a study conducted by Velazhahan et al. (2010). The reason behind is that the activity of certain plant phytochemicals like phenolics and alkaloids highly reduces upon boiling as described by Momoh et al. (2012), which is might be responsible for the alteration and breakage of the molecular structure of phytochemicals.

After detoxification, structural changes in aflatoxin molecule have been observed in several studies conducted with micro-organisms, physical and chemical agents, ultraviolet (UV) rays, Gamma rays and plant products (Alberts et al., 2006; Albores et al., 2008; Guan et al., 2010; Velazhahan et al., 2010; Wang et al., 2011; Farzaneh et al., 2012; Inoue et al., 2013; Luo et al., 2013; Samuel et al., 2014; Vijayanandraj et al., 2014). Aflatoxins have been widely researched for their toxicity by various scientists (Guengerich, 2001, 2008; Hussein and Brasel, 2001). Their toxicity data showed that aflatoxins have cyclopentene ring and furan moiety in their chemical structure. In AFB1 the presence of double bond in the terminal furan ring is key factor for its toxic and carcinogenic activities (Wang et al., 2011). In contrast, aflatoxin B2 which have a saturated furan ring is hundreds times less carcinogenic (Dvorackova, 1990). The degraded products of AFB2 may be active but were less potent than that of the parent compound. Thus, removing the double bond of terminal furan ring is a major aim of detoxification. In this present study, among six degraded products of AFB1 acquired after *T. ammi* seeds extract treatment, 50% products (with m/z 303, 341, 351) showed removal of double bond in the terminal furan ring while in products with m/z 369 and 321 both lactone group modification and double bond removal in the terminal furan ring occurred. Therefore, toxicity of most of the degraded products compared with that of aflatoxin was reduced to a much lower level.

Biological toxicity of degraded toxin products were tested using brine shrimps (*Artemia salina*) bioassay. There are several studies on the effects of aflatoxin on the brine shrimp (*Artemia salina*) eggs and larvae (Harwing and Scott, 1971; Schmidt, 1989; Logrieco et al., 1996; Durakovic et al., 2002; Moretti et al., 2007). According to previous findings (Brown, 1969; Hartl and Humpf, 2000; Favilla et al., 2006) the *Artemia salina* larvae appears to be as susceptible as biological indicator of toxicity of some mycotoxins in foods and feeds. The results of present investigation showed significant reduction in larval mortality after incubation with treated toxins as compared to untreated toxins. Similar findings were also observed by Samuel et al. (2014), who worked on detoxification of aflatoxin B1 by *Pseudomonas putida*. He compared the toxicity of treated and untreated AFB1 toward HeLa cells and concluded that degraded products are nontoxic (D1) or much less toxic (D2 and D3) than AFB1 to the cells at the tested concentrations.

From the findings of present investigation, *T. ammi* seeds extracts can be used for development of biologically safe herbal additives to food and feed products processed for human consumption to avoid the toxic effects of aflatoxins. Based on previous literature, use of ajwain in food showed no safety issues (Gemeda et al., 2014). Direct spray of aqueous plant extract is convenient for the farmers because these can be easily prepared and its application does not require any technical knowledge. However, there may be some limitations regarding formulations and shelf life of extract and research in this direction is needed.

## Author contributions

WI: PhD student who performed all the experimental work. TA: PhD supervisor, who guided and planned this project. MI: Provided expertise and equipment for mass spectroscopy. AG: Helped in data analyses. MA: provided lab facilities for high performance liquid chromatography.

## Funding

This project did not received any funding from any agency, but was run on indigenous resources of Fungal biotechnology lab, Institute of agricultural sciences, University of the Punjab, Pakistan.

### Conflict of interest statement

The authors declare that the research was conducted in the absence of any commercial or financial relationships that could be construed as a potential conflict of interest.
